# Prevalence of choroidal naevi in Germany: a cross-sectional analysis of the BiDirect study

**DOI:** 10.1186/s12886-026-05129-5

**Published:** 2026-07-17

**Authors:** Mael Lever, Alexandra Schweig, Klaus Berger, Henning Teismann, Daniela Süsskind, Marius Ueffing, Nikolaos E. Bechrakis, Andreas Stang

**Affiliations:** 1 Department of Ophthalmology, University Hospital Essen, Hufelandstr. 55, 45147 Essen, Germany; 2 Department of Ophthalmology, Tübingen University Hospital, Tübingen, Germany; 3 Institute for Ophthalmic Research, Tübingen University Hospital, Tübingen, Germany; 4 Institute of Epidemiology and Social Medicine, University of Münster, Münster, Germany; 5 Institute of Medical Informatics, Biometry and Epidemiology, University Hospital Essen, Essen, Germany

**Keywords:** Cancer surveillance, Incidence, Uveal melanoma, Choroidal melanoma, Malignant transformation, Retinal imaging, Telemedicine

## Abstract

**Background:**

Choroidal naevi (CN) are small, and mostly asymptomatic choroidal lesions. Despite their benignity, identification and follow-up of CN is required as malignant transformation is possible. CN prevalence was shown to vary greatly depending on ethnical descent, but data from European countries are rare. The aim of this study was to estimate the prevalence of CN based on fundus images from participants of the prospective German BiDirect study.

**Methods:**

Observational cross-sectional study of participants aged 37–68 years separated into three cohorts: patients with (1) depression, (2) acute cardiovascular disease, (3) participants of the general population. Participants with at least one 45°-wide macula-centred image of both eyes were included. General health indicators and eye function were assessed. The prevalence of posteriorly localised CN was calculated. Age-standardization was performed using the European Standard Population of 2013 (ESP2013) and the U.S. 2000 Standard Population (US2000). To estimate the entire fundus prevalence of CN, we applied a correction factor of 2.5 based on the assumption that 40% of all CN were detected by the study’s method. A sensitivity analysis was also performed. The association between CN presence and health indicators was investigated using logistic regression.

**Results:**

1170 participants were included. When fundus image was acquired, participants were aged 37–68 years (mean ± standard deviation: 54 ± 7.8) and 49% were females. Mean visual acuity was 0.20 ± 0.2 LogMAR on both eyes. At least one CN was detected in 36 participants. Thus, the crude prevalence of posterior CN was 3.1% (95% confidence interval: 2.1–4.1). After age-standardization, ESP2013-prevalence was 2.6% (95% CI: 2.2-3.0). A sensitivity analysis was performed to explore the potential prevalence of CN in the entire fundus; if 40% of CN were detected by our method, the entire fundus prevalence would be 7.7% (95% CI: 6.1–9.3). No association between CN and age, sex, or general health indicators was identified; the presence of CN did not impact visual acuity.

**Conclusions:**

In regions with demographics like those in the German BiDirect study, posteriorly localised CN can be expected in approximately 3.1% of the population. However, the prevalence of CN across the entire fundus remains difficult to determine.

**Supplementary Information:**

The online version contains supplementary material available at 10.1186/s12886-026-05129-5.

## Introduction

Choroidal naevi (CN) are well-delimited round or oval subretinal lesions of grey-brown or grey-green colour [1, 2]. In most cases, CN are asymptomatic and thus discovered incidentally by ophthalmoscopy [3]. They present a thickness of up to 2 mm and are sometimes associated with drusen and intraretinal fluid [4]. While benign [5], CN require regular observation since malignant transformation into choroidal melanoma, which than threatens the patient’s vision, eye, and life, is possible [6, 7]. Epidemiologic characterization of CN is therefore of clinical and socioeconomic relevance [8, 9].

CN prevalence estimations were initially conducted from autopsy eyes [10, 11]. Later clinic-based and a few population-based analyses were conducted. In general, the available population-based studies are either historical [5, 12] or based on imaging of only a small part of the fundus, which makes it difficult to estimate the overall CN prevalence [13]. Moreover, analyses from Europe are scarce and to date, only one study was conducted in Germany [14]. From these studies, disparities between the prevalence of posterior CN in populations of different ethnic origin were identified: around 2% in non-indigenous Australians [15], 2.5% in Germans [14], 5.6% in U.S. Whites and 0.6% in U.S. Blacks [13], and 1.7% to 2.9% in different Asian populations [16, 17]. The entire fundus prevalence of CN was estimated between 6.2% and 8.6% [5, 6]. Overall, comparability of these studies is complicated as the demographic characteristics of study populations vary greatly, age-standardization is often lacking, and more importantly, methods used to detect CN range from 45° fundus images to ultra-wide-field images or mydriatic fundoscopic exams.

The aim of this study was therefore to measure the prevalence of posterior CN in the German BiDirect study in order to provide more generalisable CN prevalence estimates for mainly Caucasian populations.

## Materials and methods

### Study design

The BiDirect study („Establishing the links between subclinical arteriosclerosis and depression“) is a prospective, observational, partly population-based study conducted in the region of Münster in Germany. Baseline assessments took place between 2010 and 2011 [24]. The present work consists of a cross-sectional analysis of the BiDirect study population at the time of the “second survey”, which took place two years after baseline examination. The study was conducted in accordance with the Declaration of Helsinki and written informed consent for participation in the study was obtained from all participants. The BiDirect study was approved by the ethics committee of the University of Münster, Germany and the Westphalian Chamber of Physicians (Münster, Germany). Additional approvement for the present investigation was granted by the ethics committee of the University Hospital Essen, Germany (24-12287-BO). To enhance the clarity, accuracy, and transparency of the report, this study adheres to the Strengthening the Reporting of Observational Studies in Epidemiology (STROBE) guidelines [25].

### Study population

The BiDirect study population included a total of 2258 participants aged 35–65 years at baseline and consists of three cohorts: (1) participants with a diagnosis of depression (*n* = 999), (2) participants with a history of acute coronary syndrome (*n* = 347), (3) participants from the general population (*n* = 912) [26]. Of the 1780 persons who took part in the BiDirect second survey, retinal photographs were taken from 1648 participants (92.6% of the second survey, 73.0% of the baseline study population). Subsequently, participants were excluded when image quality of one or both macula-centred photographs was insufficient for assessing the presence or absence of CN (*n* = 478, 29.0%). In the end, 1170 participants remained for the present analyses (71.0% of participants with retinal imaging; 51,8% of the whole BiDirect study population).

### Survey of general health indicators and eye function

The assessments performed at baseline and for the second survey are described in detail elsewhere [26]. A diagnostic work-up was performed including measurements of weight, height (for calculation of the body-mass-index), and waist circumference, bioelectrical impedance measurements (Body Impedance Analyzer BIA 2000-S, Data Input GmbH) including body fat and water. Blood work was also performed including blood level of glycated haemoglobin (HbA1c). Finally, the participants underwent sensory functional tests including colour vision testing using Ishihara plates and had their corrected visual acuity tested in both eyes.

### Retinal photographs acquisition and grading

**Fig. 1:**
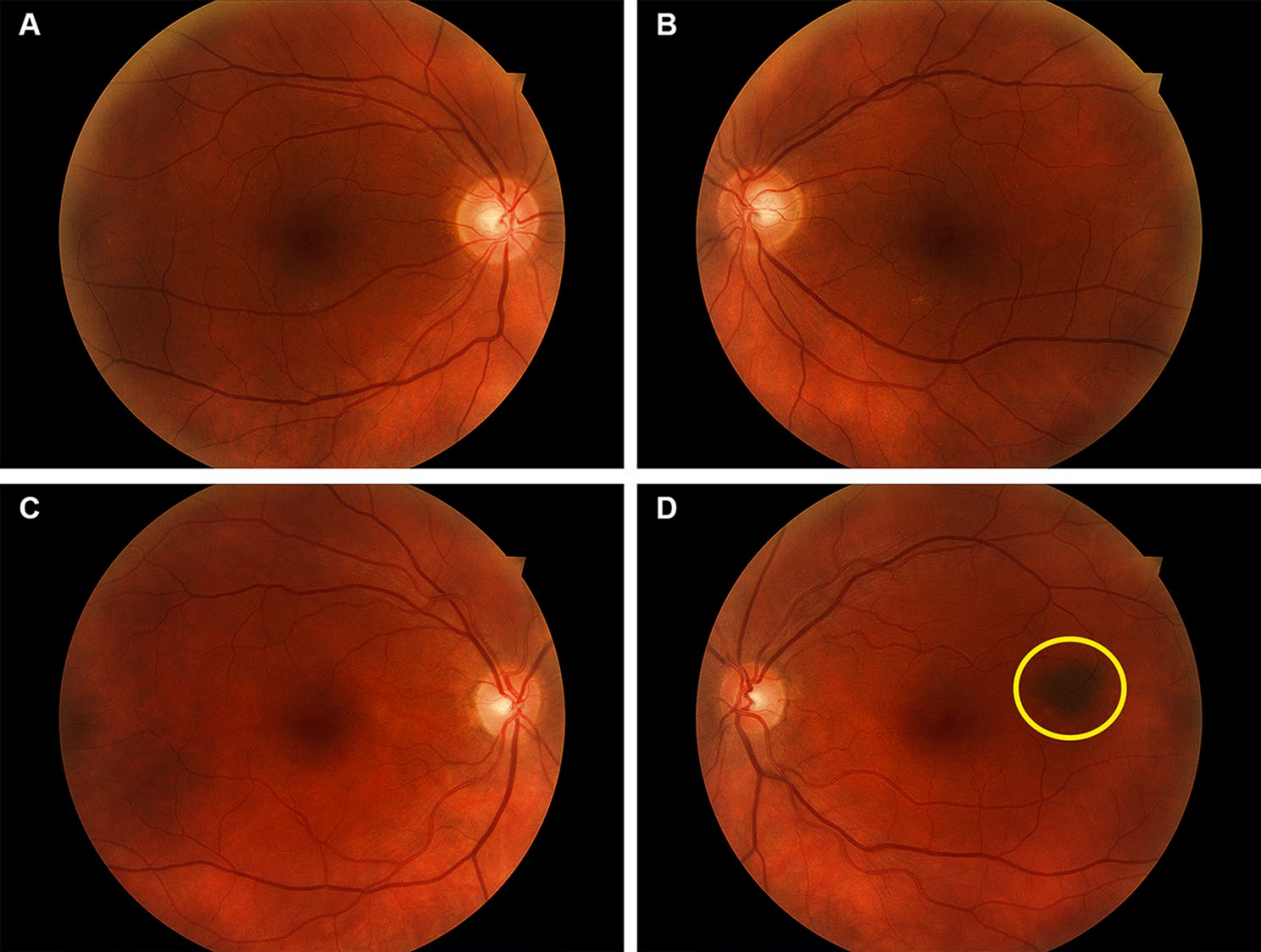
Examples of fundus images from the BiDirect study. Unremarkable fundus photograph of the right (**A**) and left (**B**) eye of a study participant. A pigmented choroidal naevus is visible in the left eye of another study participant (**D**, yellow ellipse)

During the second survey of the BiDirect study, two non-mydriatic fundus photographs were acquired from each participant’s eye, one centred on the macula and one on the optic disc. For this, the CenterView Digital Retinography System (Welch Allyn, Skaneateles Falls, NY, USA) with a 45° x 40° field of view was used. For grading, images were imported in the open-source tool Label Studio (version 1.7.1, HumanSignal, San Francisco, CA, USA). The grading workflow consisted of the following steps: the assessment of (1) image quality (good vs. insufficient), (2) right vs. left eye, (3) optic disc vs. macula centred image, (4) presence of a fundus finding, if yes (5) type of finding: CN, myelinated fibres, optic disc drusen, or other. The presence of one or more CN in each eye and the presence of CN in either or both eyes of each participant were coded binarily. CN was defined according to the criteria used in the Blue Mountain Eye Study as a homogeneous, pigmented choroidal lesion of at least 500 μm in diameter [27], still, CN of inhomogeneous appearance (e.g., presenting amelanotic areas, drusen, degeneration, or atrophy) were also classified as CN. Great care was taken to not misdiagnose other pigmented lesions like optic disc melanocytomas, pigment clumps, scars, and hypertrophies of the retinal pigment epithelium. Figure 1 shows an example of retinal images as analysed.

### Statistical analysis

Data handling was performed using Microsoft Excel (Microsoft, Redmond, WA, USA) and R with RStudio version 2024.12.0-467 (Posit Software, Boston, MA, USA). The age-truncated prevalence of CN and the corresponding 95% confidence interval was calculated. For better comparison, age-standardization was performed using both the European Standard Population of 2013 (ESP2013 [28]) and the 2000 United States standard population (US2000 [29]). Descriptive statistics include percentages and mean values ± standard deviation. To measure the potential association of CN and ophthalmic or general health parameters assessed in the BiDirect study, univariable and multivariable logistic regression was performed. Statistical analyses were performed with R in RStudio and SAS software version 9.4 for windows (SAS Institute, Cary, NC).

## Results

### Characteristics of the study population

Retinal images were available for 1648 of the 1780 participants of the second survey of the BiDirect study. After analysis of a total of 6635 images, 478 participants (29.0%) were excluded due to missing macula-centred image or insufficient quality in at least one eye, leaving 1170 participants for the final analyses including 51% men; mean age was 54.4 ± 7.8, ranging from 37 to 68 years. The percentage of men in the sub-cohorts varied with a higher percentage in the CVD sub-cohort and a lower percentage in the depression sub-cohort (Table 1). The mean corrected VA of both eyes was 0.2 ± 0.2 logMAR (equivalent to Snellen 20/32 and 0.63 decimal). For comparison, excluded participants’ characteristics are provided in Suppl. Table S1.

**Table 1:** Characteristics of men and women aged 35–65 years in the BiDirect subcohorts (Münster, Germany) 2010–2014

	All included participants	Depression sub-cohort	CVD sub-cohort	Control sub-cohort
Number of participants, (%)	1170	458 (39.1%)	181 (15.8%)	531 (45.4%)
Age, years (mean ± sd)	54.4 ± 7.8	52.6 ± 7.4	57.4 ± 6.6	55.0 ± 8.1
Male sex, n (%)	597 (51.0%)	38.6%	86.1%	49.7%
Visual acuity, logMAR				
Right eye (mean ± sd)	0.20 ± 0.20	0.21 ± 0.20	0.19 ± 0.19	0.19 ± 0.20
Left eye (mean ± sd)	0.19 ± 0.21	0.20 ± 0.20	0.19 ± 0.21	0.17 ± 0.21
BMI, kg/m^2^ (mean ± sd)	28.1 ± 5.2	29.3 ± 5.9	29.3 ± 4.2	26.7 ± 4.4

Abbreviations: BMI, body mass index; CVD, cardiovascular disease; sd, standard deviation of the mean

### Prevalence of central choroidal naevi

We detected a total of 38 CN in 38 eyes of 36 participants (20 men and 16 women). In 20 cases (55.6%), CN was in the right eye, in 14 cases (38.9%) in the left eye, and in 2 cases were bilateral (5.6%). Fourteen affected participants were in the depression sub-cohort (39%), 7 in the CVD sub-cohort (19%), and 15 in the control sub-cohort (42%). The mean age of participants with CN was 56.8 ± 6.6, ranging from 43 to 66 years. The prevalence of CN was highest (5,4%) in the 55–59 years age stratum (Table 2). VA was not influenced by the presence of a CN (mean VA for the affected right eyes 0.13 ± 0.13 logMAR, approximately Snellen 20/25, and for the affected left eyes 0.18 ± 0.19 logMAR, approximately Snellen 20/32). For this population aged 37–68 years, this relates to an age-truncated crude posterior CN prevalence of 3.1% (95% CI: 2.1–4.1). Using the ESP2013, the age-standardised prevalence was 2.6% (95% CI: 2.2-3.0), respectively 2.3% (95% CI: 1.9–2.6) using the US2000 population standard.

**Table 2:** Prevalence of choroidal nevi in the BiDirect study population stratified by age or sex

	Participants *n* (%)	Choroidal naevi *n* (%)	Prevalence (%)
Total	1170	36	3.1
Age, years			
35–39	37 (3.2%)	0 (0%)	0.0
40–44	126 (10.8%)	2 (5.6%)	1.6
45–49	192 (16.4%)	6 (16.7%)	3.1
50–54	246 (21.0%)	3 (8.3%)	1.2
55–59	242 (20.7%)	13 (36.1%)	5.4
60–64	223 (19.1%)	9 (25.0%)	4.0
65–69	104 (8.9%)	3 (8.3%)	2.9
Sex			
Male	597 (51.0%)	20 (55.6%)	3.4
Female	573 (49.0%)	16 (44.4%)	2.8

### Estimating the prevalence of choroidal naevi for the entire fundus

Using the non-mydriatic CenterView DRS camera system, only CN within or at the border of the central 45° of the posterior pole are detectable. This corresponds to approximately 2/3 of the posterior pole and 8,3% of the whole fundus surface. We performed a sensitivity analysis to explore the impact of this aspect on the potential entire fundus CN prevalence (Table 3). If CN were distributed evenly across the fundus, a correction factor of 12 would need to be applied to extrapolate from the 8.3% of the fundus visualised. Then, the entire fundus prevalence would be 36.9% (95% CI: 33.4–40.4). However, based on the spatial distribution of CN described in previous studies where the whole fundus was examined [11, 23], we hypothesised that 40% of CN were detected by our study’s approach (a justification for this choice is provided in the discussion section). Consequently, the estimated fundus-wide crude prevalence would be 7.7% (95% CI: 6.1–9.3), and 6.3% (95% CI: 5.7–7.0) after age-standardization (ESP2013), respectively 5.7% (95% CI: 5.1–6.2; US2000).

**Table 3:** Sensitivity analysis for the extrapolation of whole fundus choroidal naevus prevalence in the BiDirect study population

Estimated proportion of total choroidal naevi (%)	Correction factor	Crude prevalence (95% CI)	Age-standardised prevalence (95% CI)
Crude observation	-	3.1 (2.1–4.1)	2.5 (2.1–2.9)
8.3	12	36.9 (33.4–40.4)	30.4 (29.0–31.8)
25	4	12.3 (10.3–14.3)	10.1 (9.3–10.9)
40	2.5	7.7 (6.1–9.3)	6.3 (5.7–7.0)
50	2	6.2 (4.7–7.6)	5.1 (4.5–5.6)

This table presents several hypotheses on the proportion of total choroidal naevi (CN), that were detected by our study’s method. If CN were distributed evenly across the whole fundus, then 8.3% would have been detected by the camera system used here and a correction factor of 12 could be used to extrapolate the whole fundus CN count. The most probable assumption in our view, is that 40% of CN were detected, so that a correction factor of 2.5 can be applied. Calculations for potential whole fundus CN detection rates of 25% and 50% are also provided. Abbreviations: 95% CI, 95% confidence interval

### Association of choroidal naevi with health parameters

Using univariable logistic regression, no association between the occurrence of CN and age, body mass or height, and body mass index could be detected. Even though the odds ratios for female sex (OR = 0.83, 95% CI 0.43–1.6), HbA1c level (OR = 1.3, 95% CI 0.68–2.6), and CVD cohort (OR = 1.4, 95% CI 0.56–3.6) indicated a potential association of these parameters with the presence of CN (OR = 1.0), the precision of the estimates–as measured by the width of the 95% CI–was so low that a substantive interpretation was practically impossible. For this reason, we did not perform a multivariable logistic regression. The numerical results of the univariable logistic regression are summarised in Table 4.

**Table 4:** Association of demographic, anthropometric, and clinical parameters with the occurrence of choroidal naevi in the BiDirect study population

Parameters	OR	95% CI
Female sex	0.83	0.43–1.6
Age, years	1.2	0.99–1.5
Height, cm	1.1	0.88–1.3
Weight, kg	1.1	0.97–1.2
HbA1c, %	1.3	0.68–2.6
BMI kg/m²	1.2	0.87–1.6
CVD cohort	1.4	0.56–3.6
Depression cohort	1.1	0.52–2.3

Results of the univariable logistic regression are presented as odds ratios (OR) with their corresponding 95% confidence intervals (CI). Female sex was analysed with male sex as reference. Cardiovascular disease (CVD) and depression cohorts were compared against the control cohort as reference. Age, height, weight, body mass index (BMI), and glycated haemoglobin (HbA1c) were analysed as continuous variables; an increment of 5 units was used for all these variables but HbA1c

## Discussion

In this study, we measured the prevalence of posteriorly located (central 45°) CN in a large German study cohort. In addition to providing age-standardised rates for the age range 37–68 years, we attempted to estimate the prevalence of CN across the entire fundus.

The analysis of over 1100 bilateral macula-centred 45° fundus images from the BiDirect study-population results in an estimated crude prevalence of posteriorly located CN of 3.1% (age-standardised: 2.6%, ESP2013). This compares well with previous population-based studies of mainly white populations: the reported crude prevalence was 2.1% in Australia (general population aged 50–98 [15]) and 2.4% in New Zealand (general population aged 35–74 [30]). In the non-Hispanic white U.S. population (men and women aged 40 years and older), the crude prevalence appeared slightly higher at 4.1–5.6% [13, 31]. Regarding the German population, our age-standardised posterior CN prevalence of 2.6% (ESP2013) seems plausible as it is close to the 2.5% reported by the Gutenberg Health Study [14], which also used 45° fundus images and included participants (men and women aged 35–74 years) of the region of Mainz, in West Germany. Moreover, CN prevalence across age strata was also comparable to previous studies in Australian [5] and Asian [32] populations showing an increase from age 35 years to a peak in participants aged 50–59 years, and followed by a decrease in older patients.

This choice is based on previous studies describing the distribution of CN across the whole fundus: after anatomo-pathologic examination of enucleated eyes, Hale et al. [10] described that half CN were located equatorially and the other half near the optic disc; also in enucleated eyes, Naumann [11] observed that 2/3 of CN were located at the posterior pole; in a mydriatic fundoscopy study by Ganley and Comstock [19] 50% of CN were located posterior to the equator; in a large clinic-based study, Shields et al. [2] observed that 21% of CN were localised at the macula and 90% were posterior to the equator. More recently, Gordon-Shaag et al. [23] described that 60% of CN detected by ultra-wide field fundus imaging were located within the posterior 70° examined with the non-mydriatic camera systems of past CN prevalence studies. In general, interpretation is problematic since the definition of areas like posterior pole, equatorial region etc. vary markedly between authors. The combination of the ultra-wide field imaging study by Gordon-Shaag et al. [23] and the 1970 study by Naumann [11] were the main references for justifying our preference of correction factor, which remains subjective: to estimate the entire fundus prevalence, assuming that the camera system used would capture two thirds of the posterior pole surface and thus probable 40% of all CN, we multiplied the number of CN detected by a factor of 2.5. We also performed a sensitivity analysis to better visualise the impact of the correction factor on the potential entire fundus prevalence. Previous studies also attempted to estimate the entire fundus CN prevalence using correction factors. Based on 70° posterior pole imaging, Sumich et al. [5] assumed that 75% of all CN were detected (and thus applied a correction factor of 1.3 to their central prevalence of 6.8% to get 8.6% for the entire fundus); Qiu et al. [13] measured a central prevalence of CN in the US of 5.6% in “white” participants and argued that they would only detect 20–40% of all CN, which lead to a far higher entire fundus prevalence.

The crude entire fundus prevalence of 7.8% that we extrapolated (age-standardised with US2000: 5.7%) is in line with a pooled analysis by Singh et al. (6.2%) in the U.S [6]. but lower (8.9%) than reported from Australia [5]. Based on older anatomo-pathologic examination of autopsy eyes –where included patients should be older than the general population–, a study evaluated CN prevalence at 11% [11] and another between 9% and 20% [10]. It is difficult to assess whether these differences are genuine or the result of the CN detection methodology, given that most recent population-based studies rely on photographs of varying portions of the fundus. While clinic-based studies are more prone to selection bias (e.g., when tumour referral centres are involved) and often include a narrower age-range and fewer participants, they more frequently use indirect fundoscopy or (ultra-) wide-field imaging to detect CN and thus provide a more complete fundus examination. In general population studies of varying age ranges, the crude entire fundus CN prevalence was 4.5%, 6.2% and up to 10.9% in the U.S [18–20], and 6% in Sweden [22]. In male German military pilots, the prevalence was 4.2% [21] and 10% in Israeli students [23]. In Table 5, we provide an overview of the CN prevalence publications cited above and a more extensive list is provided in Supplementary Table S2.

**Table 5:** Selection of published prevalence estimates of choroidal naevi

Publication	Population	Age range (years)	Type of prevalence, portion of fundus	Prevalence (%)	95% CI
Hale et al. 1965	General population, autopsy based	> 18 years	Crude, complete fundus	8.6 and 20.0	4.1–13.1 and 12–28
Naumann 1970	General population, autopsy based	?	Crude, complete fundus	11.0	6.7–15.3
Ganley and Comstock 1973	Outpatients of a hospital and general population	> 30 years	Crude, complete fundus	6.2	3.4–9.0
Albert et al. 1980	White control patients	> 30 years	Crude, complete fundus	10.9	7.4–14.0
Rodriguez-Sains 1986	White control patients	11–84	Crude, complete fundus	4.5	0.6–8.6
Seregard et al. 1995	White control patients	20–80	Crude, complete fundus	6.0	1.7–10.1
Sumich et al. 1998	General population, whites	> 49	Crude, 6 × 45°	6.5	5.7–7.3
Singh et al. 2005	General population, whites	> 15	Age-standardised (US2000) complete fundus	6.2	
Ng et al. 2009	General population, Asians	40–80	Age-standardised (2000 Singapore Malay population) 6 × 45°	1.5	0.9–2.0
Greenstein et al. 2011	General population, multiethnic	45–85	Crude, 2 × 45°	4.1 in whites	3.4–4.9
Gordon-Shaag et al. 2014	Students	18–58	Crude, ultra-wide field photographs	9.6	6.7–12.5
Qiu et al. 2015	General population, multiethnic	> 40	Crude, 2 × 45°	5.6 in whites	4.8–6.7
Keel et al. 2018	General population, multiethnic	50–98	Crude, 2 × 45°	2.1 in “non-indigenous” (whites)	1.4–3.3
Elbaz et al. 2019	General population	35–74	Weighted, 30°+45°	2.4	2.1–2.7
Ramachandran et al. 2021	Diabetes mellitus patients	12–98	Crude, 45°	2.4	2.2–2.6

This table lists the published papers cited in the discussion section by year of publication, presenting the reported prevalence and its 95% confidence interval, the population under consideration (ethnicity and age range) and the proportion of the fundus examined. A more extensive list of the literature on choroidal nevi prevalence is provided in Suppl. Table S2

In addition to the limitations discussed above, additional aspects need to be mentioned. First, we can only report on age-truncated prevalence estimates as participants’ age ranged from 37 to 68 years. Furthermore, the high proportion of excluded participants due to insufficient image quality (29%) may have impacted the present results. Comparing included and excluded participants, most parameters only differ marginally (particularly sub-cohort and sex distributions, visual acuity, and BMI), however, the age difference indicates potential selection bias as the proportion of subjects 55 years and older was higher in excluded participants than in those included. CN prevalence was shown to increase gradually with age [13]; the inclusion of proportionally more younger participants (with a lower CN prevalence) and fewer older participants (with a higher CN prevalence) would both lead to an underestimation of the true prevalence. However, other studies described a different age-related prevalence trend with an increase until a peak at the age of 50–59 years followed by a decrease in older persons [5, 31, 32]. Therefore, it is difficult to estimate how much and in which direction the exclusion of elder participants would affect the results of this study. Moreover, potential age-related ocular causes of reduced image quality like media opacity, lens status, and small pupil diameter were not assessed in the BiDirect study and thus could not be used to quantify or adjust for possible selection bias. An underestimation of CN prevalence is also possible, if –particularly amelanotic– lesions were missed during the image grading process. More importantly, as with most German studies, ethnicity was not assessed in the BiDirect study. Generally, it can be assumed that almost all participants of the BiDirect study were Caucasians [33] because the proportion of citizen of countries outside Europe and North America or of inhabitants born outside of Europe is low in the Federal State of North Rhine-Westphalia, where Münster is located (4.3%, and 7.7%, respectively [34]). Consequently, multi-ethnic considerations on CN prevalence are not possible with the BiDirect study data. Finally, regarding other influencing factors, our univariable logistic regression failed to identify any association with the available health parameters, which may be due to limited statistical power caused by the low CN count in this study.

In conclusion, this cross-sectional analysis of data from a large, prospective German cohort provides new age-standardised prevalence data for choroidal naevi in an adult, mostly Caucasian population.

## Supplementary Information

Below is the link to the electronic supplementary material.

Supplementary Material 1: Supplementary Table S1. Comparison of characteristics of BiDirect study participants included or excluded from the main analyses. Abbreviations: BMI, body mass index; CVD, cardiovascular disease; sd, standard deviation of the mean; 95% CI, 95% confidence interval.

Supplementary Material 2: Supplementary Table S2. Literature overview on choroidal naevus prevalence. This table provides an extensive overview on the past and current literature on the prevalence of choroidal naevi (CN) including papers published between 1940 and December 2024 and listed in pubmed. Papers are sorted by study type (autopsy, clinic, or population-based) and the table includes the published prevalence data as well as details on the method of CN detection, the considered fundus area, the number of included subjects, the country of origine, and study-population age-range and ethnical characteristics, whenever provided.

## Data Availability

The data that support the findings of this study are available from the Institute of Epidemiology and Social Medicine, University of Münster, Münster, Germany, which conducted the BiDirect study. The data were used under a use and access permission for the current study and so are not publicly available. Data are however available from the corresponding author upon reasonable request and with permission of the Institute of Epidemiology and Social Medicine, University of Münster, Münster, Germany.

## Abbreviations

CN: Choroidal naevus/ naevi
ESP2013: European Standard Population of 2013
US2000: United States standard population of 2000
95% CI: 95% confidence interval
OR: Odds ratio
BMI: Body mass index
HbA1c: Whole blood percentage of glycated haemoglobin
