# Supplementary material for: Prevalence of choroidal naevi in Germany: a cross-sectional analysis of the BiDirect study
Source: BMC Ophthalmol. 2026 Jul 17;26:418. doi: 10.1186/s12886-026-05129-5 (PMC13386882; doi:10.1186/s12886-026-05129-5)
Supplement: Supplementary file 1 — Supplementary Material 1: Supplementary Table S1. Comparison of characteristics of BiDirect study participants included or excluded from the main analyses. Abbreviations: BMI, body mass index; CVD, cardiovascular disease; sd, standard deviation of the mean; 95% CI, 95% confidence interval. [file 12886_2026_5129_MOESM1_ESM.docx]

Supplementary Table S1. Comparison of characteristics of BiDirect study participants included or excluded from the main analyses.

|  | | **Included** **participants** | **Excluded participants** |
| --- | --- | --- | --- |
| N (%) | | 1170 (71.0%) | 478 (29.0%) |
| Sub-cohort | |  |  |
| Depression | 458 (39.1%) | 144 (30.1%) |  |
| CVD | 181 (15.8%) | 91 (19.0%) |  |
| Control | 531 (45.4%) | 243 (50.3%) |  |
| Male sex, n (%) | | 597 (51.0%) | 257 (53.7%) |
| Age (mean ± sd) | | 54.4 ± 7.8 | 57.7 ± 7.2 |
| 35-39 years, n (%) | | 37 (3,2%) | 5 (1.0%) |
| 40-44 years, n (%) | | 126 (10,8%) | 28 (5.9%) |
| 45-49 years, n (%) | | 192 (16,4%) | 46 (9.6%) |
| 50-54 years, n (%) | | 246 (21.0%) | 78 (16.3%) |
| 55-59 years, n (%) | | 242 (20,7%) | 107 (22.4%) |
| 60-64 years, n (%) | | 223 (19,1%) | 128 (26.8%) |
| 65-69 years, n (%) | | 104 (8,9%) | 86 (18.0%) |
| Visual acuity, logMAR | |  |  |
| Right eye (mean ± sd) | | 0.20 ± 0.20 | 0.22 ± 0.24 |
| Left eye (mean ± sd) | | 0.19 ± 0.21 | 0.20 ± 0.21 |
| BMI kg/m^2^, (mean ± sd) | | 28.1 ± 5.2 | 28.0 ± 4.9 |

Abbreviations: BMI, body mass index; CVD, cardiovascular disease; sd, standard deviation of the mean.
